# Decision-centered wearable biosensors for personalized rehabilitation: integrating multimodal monitoring, artificial intelligence, and closed-loop intervention

**DOI:** 10.3389/fbioe.2026.1948186

**Published:** 2026-09-18

**Authors:** Fujin Jia, Xinzhu Li, Hui Song, Xinyue Liu, Yuli Tang, Qingping Wen, Ping Wu

**Affiliations:** 1 College of Health-Preservation and Wellness, Dalian Medical University, Dalian, China; 2 Department of Anesthesiology, The First Affiliated Hospital of Dalian Medical University, Dalian, China

**Keywords:** artificial intelligence, closed-loop intervention, digital biomarkers, edge computing, multimodal sensor fusion, personalized rehabilitation, real-time biofeedback, wearable biosensors

## Abstract

Personalized rehabilitation requires repeated assessment of movement, physiological tolerance, fatigue, and adherence, yet conventional clinic-based measurements provide only intermittent snapshots. The convergence of wearable biosensors with artificial intelligence, edge-cloud computing, and connected data infrastructures creates an opportunity to convert continuous multimodal signals into timely rehabilitation decisions. This critical narrative review synthesizes physiological, biochemical, electrophysiological, biomechanical, and behavioral sensing within a monitoring–interpretation–feedback–adaptation–outcome framework. We first evaluate analytical validity, real-world robustness, biocompatibility, wearability, calibration drift, and power continuity. We then examine signal-quality control, multimodal fusion, digital biomarkers, federated learning, digital twins, and predictive models according to latency, uncertainty, generalizability, privacy, and decision utility. The review follows biosensor-derived information across a graded spectrum of action, from clinician-reviewed monitoring and real-time biofeedback to assist-as-needed adaptation, functional electrical stimulation, and state-responsive neuromodulation. Applications include motor and physiological assessment, fatigue-aware dose adjustment, musculoskeletal and neurological rehabilitation, posture correction, sensor-rich prosthetics, and Internet of Things–enabled home rehabilitation. Across these settings, greater autonomy increases the evidence and safety requirements: models should expose confidence, systems should degrade safely when signal quality falls, and digital infrastructures should preserve interoperability, version traceability, privacy, and accountable clinician oversight. The central translational gap is not sensor sensitivity alone but whether an integrated biosensor system produces a reliable metric, supports an explicit and proportionate decision, and improves an outcome important to the patient. Decision-centered evaluation can help move wearable biosensing from fragmented technology demonstrations toward secure, personalized, continuous, and clinically accountable rehabilitation.

## Graphical Abstract

**Figure:**
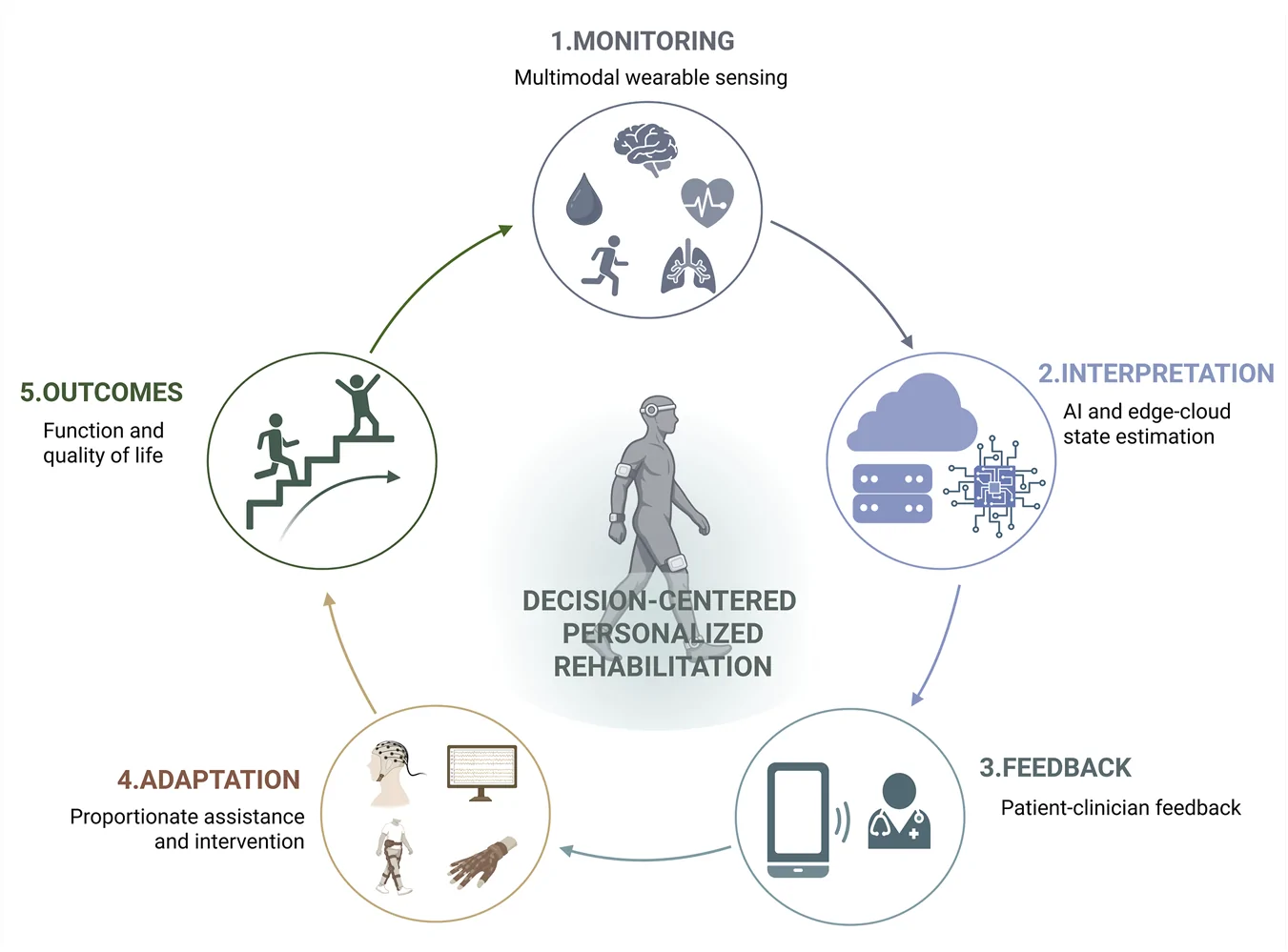
A decision-centered closed-loop wearable biosensing framework for personalized rehabilitation. Created by the authors.

## 1 Introduction

Population aging and the growing burden of neurological, cardiopulmonary, and musculoskeletal disorders are increasing demand for rehabilitation services worldwide. By 2050, people aged 60 years or older are projected to account for 22% of the global population (World Health Organization, 2025). This transition is occurring alongside persistent constraints in rehabilitation capacity, creating pressure for care models that extend beyond episodic hospital visits toward individualized, longitudinal, and home-centered rehabilitation (Bakhshayesh et al., 2025). Conventional treatment progression is still commonly guided by brief clinic-based observations and semi-quantitative scales. Although tools such as the Modified Ashworth Scale remain clinically useful, their reliability varies with muscle group, examiner experience, and testing protocol (Pinho et al., 2024; Harb et al., 2025). Intermittent assessment cannot fully capture day-to-day changes in movement quality, physiological tolerance, fatigue, adherence, or real-world activity; rehabilitation dose and progression may therefore lag behind a patient’s changing capacity.

Wearable biosensors and complementary body-worn sensors can address this gap by enabling continuous, context-rich monitoring of physiological, biochemical, electrophysiological, biomechanical, and behavioral signals. When technically valid and clinically interpretable, these data can become digital biomarkers that complement clinic-based assessments with information about frequency, intensity, symmetry, variability, and real-world performance (Daniore et al., 2024; Cai et al., 2025). Artificial intelligence (AI), edge computing, and connected platforms can further support risk detection, movement classification, recovery forecasting, and individualized adjustment of rehabilitation plans (Goldsack et al., 2020; Zadka et al., 2024). The central opportunity is therefore not simply to collect more data, but to link an integrated biosensor measurement to a specific action: changing exercise intensity, correcting movement strategy, progressing loading, altering assistance, triggering stimulation, or requesting clinician review (Rebelo et al., 2023).

In this review, wearable biosensing refers to body-worn platforms that directly measure biological or physiological variables—such as electromyography (EMG), electrocardiography or photoplethysmography, respiration, temperature, neural activity, and sweat chemistry—together with complementary biomechanical transducers, including inertial, pressure, strain, and force sensors. The distinction is important: an inertial sensor alone is not a biosensor, but its integration with physiological or biochemical sensing can provide the movement context required to interpret biological signals and support rehabilitation decisions.

Existing reviews often address flexible biosensors, wearable validation, AI, rehabilitation robotics, or neuromodulation as separate domains (Lu et al., 2020; Jia, 2022; Molle et al., 2026). This fragmentation obscures the system-level dependencies that determine clinical value: signal quality constrains model confidence; synchronization, connectivity, and latency constrain feedback timing; biocompatibility and wearability affect adherence and data completeness; and automatic adaptation increases the consequences of sensor, software, or communication failure. A decision-centered perspective is therefore needed to connect biosensor design and digital integration with interpretation, feedback, adaptation, and clinically meaningful outcomes.

We organize integrated wearable rehabilitation around a five-stage monitoring–interpretation–feedback–adaptation–outcome framework. Monitoring asks whether physiological and biomechanical signals are valid, reliable, and sustainable during real use. Interpretation includes signal-quality control, multimodal fusion, uncertainty, and generalizability. Feedback concerns whether information reaches the right person at the right time and in a form that supports self-correction or clinician action. Adaptation concerns safe changes in exercise dose, task difficulty, assistance, or stimulation. Outcome evaluation closes the loop by determining whether these adjustments improve function, participation, adherence, safety, and quality of life. Cross-cutting requirements include biocompatibility, power continuity, secure data transmission, interoperability, version traceability, clinician oversight, ethical governance, regulatory readiness, and fail-safe degradation.

The cross-cutting principles governing feedback, adaptation, and outcome reassessment are summarized in Box 1. These principles represent an author-proposed synthesis of established rehabilitation, motor-learning, biosensor-validation, and safety principles, rather than a previously validated framework.

Box 1 **Decision principles for wearable closed-loop rehabilitation.**

Task-specific practice and active participation. Wearable systems should increase meaningful, goal-directed practice without replacing voluntary effort. Progression should be evaluated through active repetitions, unassisted performance, and transfer to relevant daily activities (Levin and Demers, 2021).

Timely and proportional feedback. Visual, auditory, haptic, or stimulation-based feedback should be temporally linked to the movement or physiological state being modified. Feedback frequency and intensity should be sufficient to support correction while avoiding overload, habituation, and dependence (Muthukrishnan et al., 2019).

Assist-as-needed adaptation. Exercise dose, task difficulty, mechanical assistance, and stimulation should adapt to performance and tolerance while preserving patient effort. Adaptation should remain within predefined biomechanical, physiological, pain, and stimulation safety limits (Hsu et al., 2024).

Confidence- and fatigue-aware control. Signal quality, model confidence, fatigue, pain, and physiological tolerance should be assessed before an automated adjustment is applied. When evidence becomes unreliable or safety limits are approached, the system should reduce adaptation, request clinician review, or enter a safe state (Borda et al., 2023; Daniore et al., 2024).

Outcome reassessment. Immediate device-assisted improvement should be distinguished from durable rehabilitation benefit. Systems should reassess retention after feedback or assistance withdrawal, transfer to unassisted activities, adherence, participation, safety, function, and quality of life, and use these outcomes to update subsequent feedback or adaptation (Goldsack et al., 2020; Levin and Demers, 2021).

This narrative review makes four contributions. First, it maps integrated physiological, biochemical, electrophysiological, and biomechanical sensing according to the rehabilitation variables and decisions each modality can support, rather than treating analytical sensitivity as an end point. Second, it evaluates material compatibility, wearability, interface reliability, power endurance, and signal quality as determinants of continuous monitoring. Third, it connects AI, edge-cloud processing, multimodal fusion, and personalized prediction with real-time biofeedback, assist-as-needed adaptation, functional electrical stimulation (FES), and neuromodulation across a graded spectrum of closed-loop autonomy. Fourth, it examines how longitudinal biosensor data can support home rehabilitation and individualized care while remaining accountable to privacy, interoperability, safety, regulatory, and outcome evidence.

The practical question throughout the review is whether a wearable measurement changes a rehabilitation decision and whether that decision improves an outcome that matters to the patient. A sweat lactate signal is useful only if it can reliably inform exercise-intensity review; an inertial measurement unit (IMU)-derived gait metric is useful only if it supports movement correction, progression, or safety assessment; and a neural-decoding system is useful only if stimulation is triggered with adequate confidence, timing precision, and fail-safe control.

## 2 Methods

### 2.1 Literature screening strategy

This narrative review was conducted using a transparent literature identification and selection approach guided by the principles of PRISMA 2020 (Preferred Reporting Items for Systematic Reviews and Meta-Analyses) (Page et al., 2021). Although this review was not designed as a systematic review or meta-analysis, PRISMA principles were adopted to improve transparency in reporting the literature sources, search strategy, and study selection process.

A comprehensive literature search was performed in PubMed, Web of Science, Scopus, and IEEE Xplore for studies published from 1 January 2017, to 1 July 2026. The search strategy combined controlled vocabulary and free-text terms covering four major concepts: (1) wearable biosensors and multimodal sensing, including physiological, biochemical, electrophysiological, and biomechanical monitoring; (2) artificial intelligence and digital technologies, including machine learning, multimodal fusion, digital biomarkers, edge computing, and digital twins; (3) rehabilitation feedback and adaptive intervention, including biofeedback, closed-loop control, functional electrical stimulation, and neuromodulation; and (4) personalized, remote, and home-based rehabilitation applications. The detailed search strategy and Boolean logic are presented in Table 1, with PubMed search syntax used as a representative example.

**TABLE 1:** PubMed database search strategy.

Number	Search strategy
#1	“Rehabilitation”[MeSH Terms]
#2	“Rehabilitation”[Title/Abstract]
#3	#1 OR #2
#4	“Wearable Electronic Devices”[MeSH Terms]
#5	((((Wearable Electronic Device*[Title/Abstract]) OR (Wearable Device*[Title/Abstract])) OR (Wearable Technology*[Title/Abstract])) OR (Electronic Skin[Title/Abstract])) OR (Wearable Computer*[Title/Abstract])
#6	#4 OR #5
#7	“Artificial Intelligence”[MeSH Terms]
#8	((((((Artificial Intelligence[Title/Abstract]) OR (Computer Reasoning[Title/Abstract])) OR (Machine Intelligence[Title/Abstract])) OR (Computational Intelligence[Title/Abstract])) OR (Computer Vision System*[Title/Abstract])) OR (Knowledge Acquisition[Title/Abstract])) OR (Knowledge Representation*[Title/Abstract])
#9	#7 OR #8
#10	#3 AND #6 AND #9

PubMed search syntax is presented as a representative example of the literature search approach, with database-specific adaptations applied when necessary.

Additional relevant publications were identified through manual screening of reference lists from key studies and reviews. The retrieved literature was qualitatively synthesized according to the contribution of wearable biosensing systems to monitoring, interpretation, feedback, adaptation, and rehabilitation outcomes. Given the substantial heterogeneity in technologies, study designs, populations, and outcomes, no formal risk-of-bias assessment or quantitative synthesis was performed. Instead, evidence maturity was qualitatively classified according to validation stage, ranging from laboratory validation and technical feasibility to field validation, clinical feasibility, controlled evaluation, and real-world implementation.

### 2.2 Inclusion and exclusion criteria

#### 2.2.1 Inclusion criteria

Publications were considered eligible when they addressed one or more of the following aspects: (1) wearable biosensors or relevant body-worn sensing technologies capable of acquiring physiological, biochemical, electrophysiological, biomechanical, or behavioral signals; (2) integration of wearable-derived information with artificial intelligence, computational analysis, multimodal data fusion, or digital healthcare platforms; (3) applications related to biomedical monitoring, personalized rehabilitation, functional assessment, remote rehabilitation, or rehabilitation decision support; and (4) evidence contributing to understanding the technical development, clinical relevance, or translational potential of wearable sensing systems in rehabilitation.

#### 2.2.2 Exclusion criteria

Publications were excluded when they were considered beyond the scope of this review, including: (1) studies with inconsistent publication types or insufficient relevance to the review topic; (2) studies not focusing on wearable biosensing or relevant body-worn sensing technologies; (3) studies unrelated to biomedical monitoring, rehabilitation applications, or rehabilitation decision-making; (4) materials or device-oriented studies without a plausible connection to rehabilitation applications; and (5) theoretical or computational studies lacking experimental or real-world validation.

### 2.3 Study screening process

Retrieved publications were screened based on their relevance to the objectives of this narrative review. After removal of duplicate records, titles and abstracts were initially reviewed, followed by full-text assessment of potentially relevant studies. The overall literature identification and selection process is summarized in Figure 1.

**FIGURE 1:**
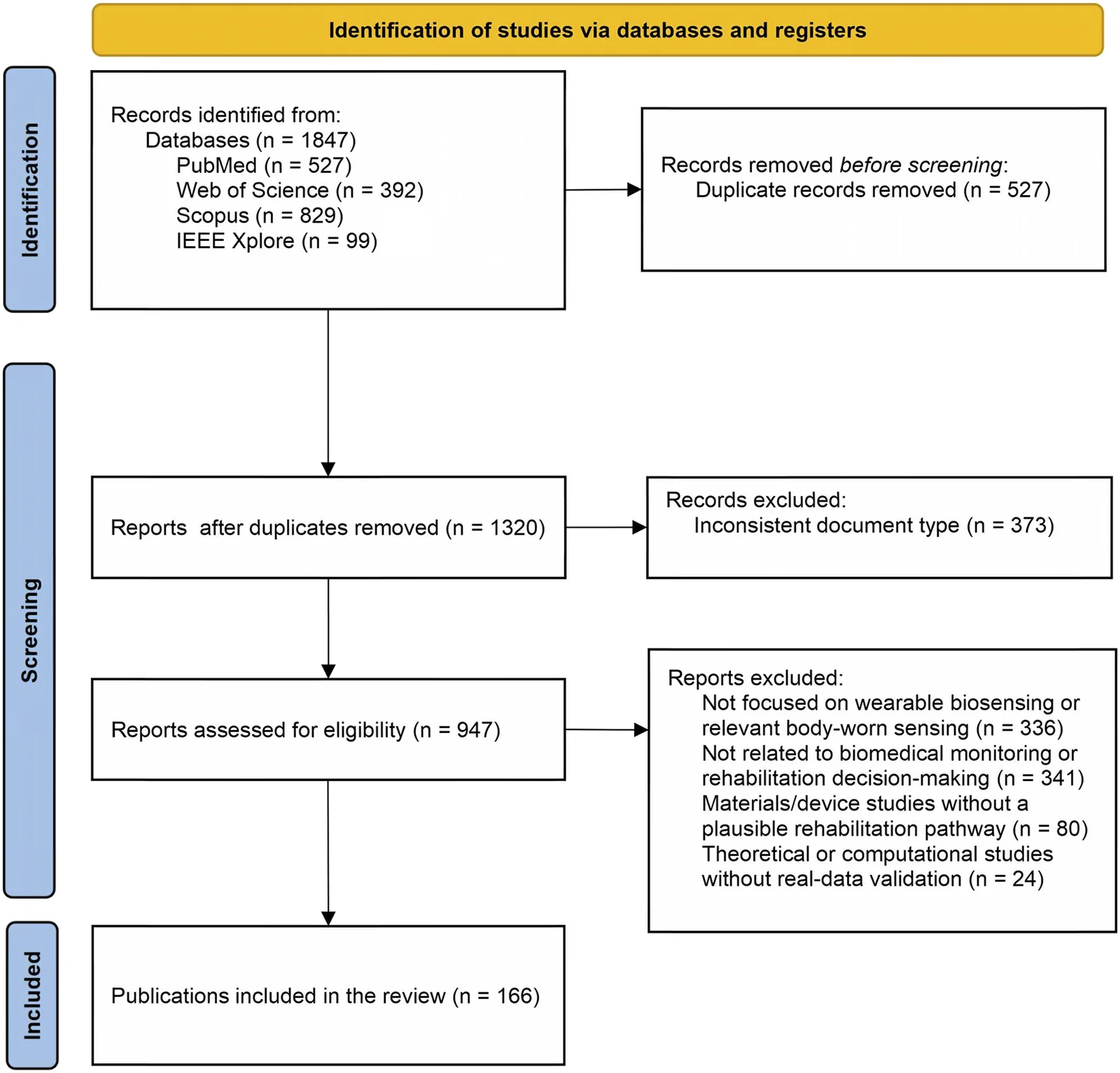
Flow diagram of literature identification and screening.

During study selection, particular attention was given to whether wearable sensing systems provided meaningful biological or functional information, whether computational approaches enabled effective interpretation of multimodal data, and whether the generated information could contribute to rehabilitation monitoring, feedback, adaptation, or decision-making. This approach reflects the decision-centered perspective of this review, which emphasizes the transition from wearable biosensor measurements toward clinically meaningful rehabilitation applications.

The final selected publications provided the foundation for qualitative synthesis, while additional background literature related to biosensor technologies, artificial intelligence, rehabilitation science, and digital healthcare infrastructure was incorporated to support conceptual interpretation and discussion.

## 3 Integrated wearable biosensors for personalized rehabilitation

Integrated wearable rehabilitation depends on coordinated biosensing, complementary movement sensing, reliable human–device interfaces, continuous power, and decision-oriented computation. Biosensors determine which biological states can be measured; biomechanical transducers provide task and movement context; material and interface stability determine whether signals remain trustworthy over time; and edge-cloud software determines whether heterogeneous streams can be translated into feedback or adaptation. The following sections move from multimodal biosensing and longitudinal analytical reliability to AI-enabled interpretation and distributed decision support (Lu et al., 2020; Iqbal et al., 2021; Priyadarshi et al., 2026).

### 3.1 Multimodal biosensing and analytical reliability

Wearable platforms increasingly combine physiological, biochemical, kinematic, kinetic, and electrophysiological signals (Cheng et al., 2025; Yao et al., 2025). Their value lies in complementary views of rehabilitation state: physiological and biochemical sensors describe internal load and tolerance; movement sensors describe task exposure and movement quality; EMG and neural signals describe activation and intention; and pressure or strain sensors characterize body–environment interaction. Each modality introduces trade-offs among analytical validity, selectivity, comfort, calibration burden, power consumption, and long-term stability.

#### 3.1.1 Multimodal physiological, biochemical, and biomechanical sensing

The value of an integrated wearable platform begins with signal validity under the conditions in which rehabilitation actually occurs. As illustrated in Figure 2, connected data acquisition and analytics (A) integrate data from exercise-worn electrochemical sensors (B), wrist-mounted thermoelectric sensors (C), mask-integrated flexible gas sensors (D), body-worn IMUs (E), and force-sensing insoles (F), with wireless transfer to mobile devices, cloud services, and workstation-based analytics. Sensitivity and limit of detection are important but insufficient. Selectivity, motion-artifact resistance, drift, response time, calibration burden, synchronization, and stability during prolonged wear determine whether a signal can support a decision outside the laboratory (Gao et al., 2023; Davis et al., 2024; Yin et al., 2024). A defensible design strategy is to combine the smallest set of complementary signals required to estimate internal load, movement quality, adherence, and recovery while limiting user burden and redundant data collection.

**FIGURE 2:**
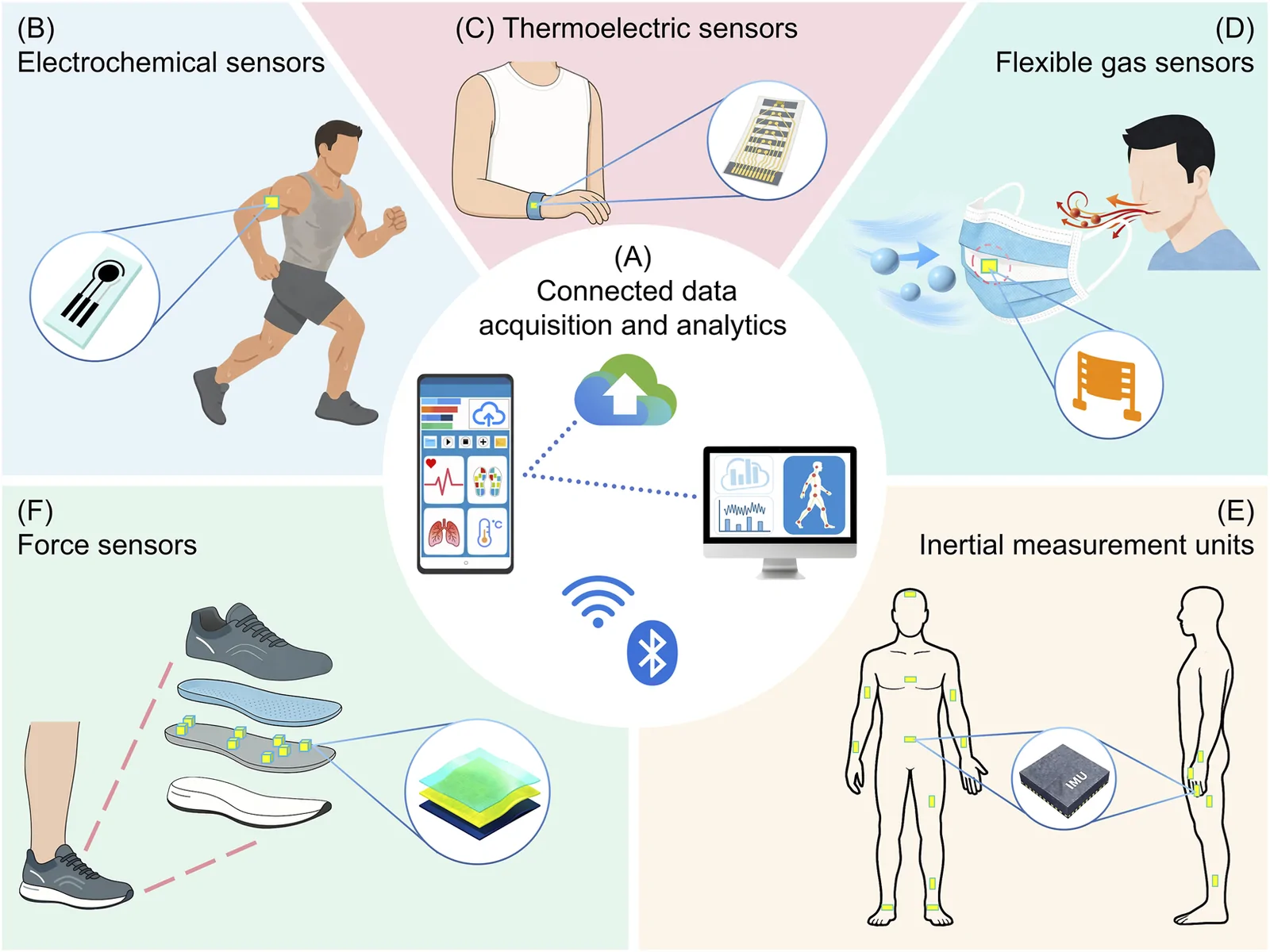
Connected multimodal wearable sensing architecture for rehabilitation monitoring. **(A)** Wireless data acquisition and analytics across mobile, cloud, and workstation platforms. Created by the authors. **(B)** Exercise-worn electrochemical sensors for metabolic-marker monitoring to assess exercise tolerance and recovery (Gao et al., 2023). Adapted from (Ma L. et al., 2024) Copyright 2024 CC BY 4.0. **(C)** Wrist-mounted thermoelectric sensors for skin-temperature, perfusion, and pulse-related monitoring. Adapted from (Tian et al., 2026) Copyright 2026 CC BY 4.0. **(D)** Mask-integrated flexible gas-sensing platform for continuous respiratory-cycle monitoring (Kim D. et al., 2024; Song et al., 2024; Jannat et al., 2025). Adapted from (Xu et al., 2025) Copyright 2025 CC BY 4.0. **(E)** Body-worn inertial measurement units for posture and movement monitoring (Salchow-Hömmen et al., 2022; Weizman et al., 2022). Created by the authors. **(F)** Multilayer insole integrating distributed force sensors for plantar loading assessment (Duong et al., 2023; Jin et al., 2026). Created by the authors.

##### 3.1.1.1 Biochemical and physiological biosensing

Biochemical and physiological biosensors extend rehabilitation monitoring beyond visible movement. Wearable electrochemical devices can quantify lactate, glucose, and related metabolic markers, potentially complementing heart rate and perceived exertion when estimating exercise tolerance or recovery (Ma J. et al., 2024; Chen Y. et al., 2025). Flexible thermal, vascular, and respiratory sensors can add information on skin temperature, perfusion, airflow, humidity, and breathing patterns (Tang et al., 2023; Kim D. et al., 2024; Song et al., 2024; Harun-Or-Rashid et al., 2025; Jannat et al., 2025; Zhang et al., 2025; Tian et al., 2026). Their clinical value depends on interpretability in real samples and changing contexts. Sweat rate, local sampling, environmental temperature, biofouling, humidity, and sensor aging can alter the relationship between the measured signal and systemic state; a low detection limit should therefore not be equated with clinical actionability.

Multimodal physiological platforms are increasingly integrated into wristbands, masks, textiles, and epidermal patches. Thermal arrays can capture arterial pulsation, perfusion trends, skin temperature, and heart rate–related information, whereas wearable respiratory sensors can characterize inhalation–exhalation patterns (Kim D. et al., 2024; Song et al., 2024; Jannat et al., 2025; Tian et al., 2026). For rehabilitation, the priority is not maximal channel count but reliable detection of meaningful changes in tolerance or safety with acceptable false-alarm rates, calibration stability, biocompatibility, and user burden during repeated sessions and home use.

Movement-oriented rehabilitation additionally requires reliable measurement of kinematics, muscle activation, and interaction forces.

##### 3.1.1.2 Kinematic, kinetic, and electrophysiological sensing

IMUs combine accelerometers, gyroscopes, and sometimes magnetometers to measure posture, gait, and task-specific movement, while pressure, strain, and force sensors quantify plantar loading, center-of-pressure trajectories, limb–environment interaction, and weight-bearing progression (Kim et al., 2021; North et al., 2024; Salaorni et al., 2024). Surface EMG adds biological information on activation timing, co-contraction, and fatigue. The main limitation is reliability under unconstrained use: IMUs can drift, soft sensors can show hysteresis and sweat ingress, EMG is sensitive to electrode placement and motion artifact, and asynchronous clocks can distort the relationship among gait phase, plantar load, muscle activation, and physiological response. Multimodal streams therefore require synchronization, signal-quality gating, and calibration procedures that are practical enough to repeat across sessions.

#### 3.1.2 Wearability, biocompatibility, and long-term signal fidelity

Longitudinal rehabilitation requires sensors that remain comfortable, biocompatible, and analytically stable during repeated movement, sweating, donning and doffing, and home use. Mechanical mismatch at the skin–device interface can increase discomfort, motion artifact, and electrode-impedance variability (Yin et al., 2024; Zhao et al., 2024). Conformal electronic skin, soft electrodes, electronic textiles, and strain-decoupled interconnects are clinically relevant not because flexibility is intrinsically desirable, but because interface stability affects data completeness, signal quality, and adherence (Cho et al., 2022; Li et al., 2022; Tian et al., 2024).

Functional materials including MXene composites, graphene and carbon-nanotube networks, liquid metals, conductive elastomers, and hydrogels can provide high stretchability and sensitivity (Orts Mercadillo et al., 2022; Chae et al., 2023; Liu et al., 2024; Zhan et al., 2026). Their translational value, however, is determined by whether performance survives humidity, oxidation, repeated deformation, and manufacturing variation. For example, highly stretchable sensors may still produce clinically misleading joint-angle estimates if viscoelastic hysteresis or inter-device variability is not controlled. Material selection should therefore be evaluated through the rehabilitation task and expected wear environment rather than by peak sensitivity alone.

Interface engineering is equally important. Breathable nanomeshes and porous elastomers can reduce sweat accumulation; biocompatible encapsulation can isolate potentially harmful components; and self-healing or tissue-like hydrogels can maintain low interfacial impedance during deformation (Usman et al., 2023; Dong et al., 2024; Alagarsamy et al., 2025; Kazanskiy et al., 2025). These features should be assessed together with skin tolerance, adhesion, signal-to-noise ratio, and recalibration frequency during extended wear. Comfort and analytical performance are coupled outcomes: poor breathability changes impedance and biochemical background signals, whereas poor adhesion increases motion artifact and missing data.

At the device level, electronic textiles can extend EMG or pressure monitoring over larger body regions, while kirigami and serpentine interconnects mechanically isolate rigid chips from joint deformation (Tian et al., 2024; Yang et al., 2024; Zhu L. et al., 2025). Such form-factor innovations are most useful when they simplify placement, reduce donning time, survive cleaning, and maintain reproducible sensor location across sessions.

The field should move from maximizing isolated laboratory performance toward standardized tests of rehabilitation robustness. Relevant evaluation includes repeated donning and doffing, washing or cleaning, skin motion, temperature change, mechanical shear, sweat exposure, day-to-day recalibration, and testing in target populations rather than only healthy volunteers. Piezoresistive hysteresis, biochemical biofouling, hydrogel dehydration, electrode displacement, and aging can all undermine a metric that appeared accurate in a short bench test (Hou et al., 2025; Jin et al., 2026; Zeng et al., 2026). Table 2 summarizes wearable sensing modalities in terms of signals, derived metrics, supported decisions, and validity constraints, underscoring that modality selection should be matched to the intended rehabilitation decision rather than isolated technical performance.

**TABLE 2:** Wearable sensing modalities for rehabilitation: signals, derived metrics, supported decisions, and validity constraints.

Sensing modality	Representative signals	Rehabilitation metric	Decision supported	Key validity constraints
IMU or accelerometry (Weizman et al., 2022; Dabnichki and Pang, 2024; Salaorni et al., 2024)	• Acceleration • Angular velocity • Orientation	• Gait phase and symmetry • Gait variability • Activity exposure	• Movement correction • Rehabilitation progression • Fall-risk review	• Drift and placement sensitivity • Generalizability to pathological movement
EMG or electrophysiology (Zhang et al., 2023; Dabnichki and Pang, 2024; Jin et al., 2024)	• Muscle activation • Activation timing • Neural activity	• Activation timing • Co-contraction and fatigue • Motor intention	• Biofeedback • Exercise selection • Assistance or stimulation timing	• Motion and stimulation artifacts • Electrode placement and impedance variability
Pressure, strain, or force sensing (Duong et al., 2023; North et al., 2024; Jin et al., 2026)	• Plantar pressure • Contact force • Joint deformation	• Loading symmetry • Center of pressure • Range of motion	• Weight-bearing progression • Movement correction • Return-to-function review	• Hysteresis and repeated-loading drift • Sweat ingress and calibration burden
PPG, ECG, or respiratory biosensing (Bayoumy et al., 2021; Liu et al., 2025)	• Heart rate and rhythm • Oxygenation • Breathing pattern	• Physiological tolerance • Internal load • Recovery trend	• Exercise-intensity adjustment • Safety alerts • Clinician escalation	• Motion and contact sensitivity • Context-dependent interpretation
Biochemical or thermal biosensing (Davis et al., 2024; Ma et al., 2024a; Tian et al., 2026)	• Lactate and glucose • Sweat chemistry • Skin temperature	• Metabolic state • Physiological context • Environmental context	• Adjunctive intensity guidance • Recovery review • Safety review	• Sampling and matrix-dependent calibration • Biofouling and environmental confounding

Abbreviations: ECG, electrocardiography; EMG, electromyography; IMU, inertial measurement unit; PPG, photoplethysmography.

IMUs are well suited to movement monitoring but require drift and placement control; pressure and strain sensors quantify loading but may show hysteresis; EMG provides neuromuscular information but is vulnerable to placement and motion artifacts; and biochemical biosensors provide molecular specificity but require real-matrix calibration and anti-biofouling strategies. The preferred platform is the smallest sensor set that provides sufficient accuracy, complementary information, redundancy, and user acceptability for the intended decision.

A central translational principle follows: wearability, biocompatibility, and signal quality are coupled requirements. Poor breathability, unstable adhesion, excessive stiffness, or difficult placement can degrade both adherence and measurement validity (Goyal et al., 2022; Davis et al., 2024; Yin et al., 2024). Long-term studies should therefore evaluate comfort, skin safety, missing-data patterns, signal drift, and stability of the derived rehabilitation metric as interdependent outcomes.

#### 3.1.3 Power endurance and continuity of monitoring

Power endurance is a continuity-of-care problem as much as an engineering problem. Frequent charging, bulky batteries, and unexpected shutdowns create missing data and reduce adherence during sleep, outdoor activity, and home training (Yin and Wang, 2023). Mechanical, photovoltaic, thermoelectric, hydrovoltaic, and biofuel cell harvesters can supplement low-power sensing or standby functions (Das et al., 2023; Tabaie and Omidvar, 2023; Cao et al., 2024; Shi et al., 2024; Su et al., 2024; Zhu J. et al., 2025), but output is intermittent and environment dependent. This limitation is particularly relevant in rehabilitation because people with severe motor impairment may generate the least harvestable mechanical energy.

A realistic power budget view is therefore preferable to claims of complete energy autonomy. Biomechanical and thermoelectric harvesters commonly operate below the power required for continuous multimodal sensing, wireless streaming, or active FES and robotic actuation (Tosi et al., 2017; Burton et al., 2023; Peng et al., 2023). Fully battery-free active rehabilitation remains unrealistic for many use cases (Qu et al., 2026). Practical systems should combine rechargeable storage, duty-cycle optimization, selective edge inference, low-power communication, and auxiliary harvesting so that monitoring remains continuous and high-power intervention is available when required (Kang and Yeo, 2024). The relevant outcome is system availability: whether the sensing–feedback–intervention chain is operational when a clinically meaningful event occurs.

Wireless power transfer can complement onboard storage for selected skin-mounted or implantable systems (Yahya Alkhalaf et al., 2022). Long-term use nevertheless requires evaluation of electromagnetic compatibility, skin heating, charging ergonomics, and failure behavior. For most wearable rehabilitation systems, the near-term goal is not battery elimination but reducing charging burden and preventing power interruptions from breaking the monitoring–feedback–adaptation loop.

### 3.2 AI and distributed computing for decision support

Digital integration determines whether wearable signals become actionable information. A defensible computational pipeline must first detect poor-quality data, synchronize heterogeneous channels, and extract task-relevant features; it must then estimate state or risk with calibrated confidence; finally, its output must map to an explicit feedback or adaptation rule. The following sections examine edge-cloud processing and multimodal fusion, personalized prediction and safe adaptation, and biosensor-enabled biofeedback. In rehabilitation, a software error is not merely a classification error when it can propagate into inappropriate coaching, excessive assistance, or unsafe stimulation.

#### 3.2.1 Edge-cloud processing, data quality, and multimodal fusion

Wearable terminals generate heterogeneous data streams that require preprocessing before they can support decisions. Cloud platforms provide storage and retrospective population-level analysis, but variable latency and continuous bandwidth requirements are unsuitable for time-critical feedback or therapeutic actuation (Islam et al., 2025; Xi et al., 2025). Edge computing places inference on local processors, enabling rapid responses for fall detection, gait-phase recognition, exoskeleton control, and FES triggering (Krasteva et al., 2025). Embedded feasibility remains constrained by memory and power budgets (Fanariotis et al., 2023). Motion-artifact removal, adaptive filtering, sensor synchronization, task segmentation, normalization, and signal-quality estimation are therefore determinants of reproducibility and downstream decision validity.

At the system level, continuous rehabilitation monitoring is an Internet of Things architecture rather than a stand-alone sensor. Local devices should preserve time-critical functions and a safe fallback state, while secure cloud services support longitudinal storage, population analytics, clinician dashboards, and non-urgent model updates (Islam et al., 2025; Xi et al., 2025). The architecture should report connectivity loss, clock drift, software and firmware versions, calibration state, and data provenance so that an apparent physiological change can be distinguished from a technical change in the sensing pipeline.

Algorithms should be co-designed with the latency requirement of the loop they serve. Quantization, pruning, and related compression strategies can reduce memory and energy use (Kasper et al., 2025), while neuromorphic and spiking approaches offer event-driven low-power inference (Alinsaif, 2026). A posture cue or clinician dashboard can tolerate longer delays than a stimulation trigger designed to pair motor intention with afferent feedback. For associative neuromodulation, timing requirements should be reported against paradigm-specific physiological windows (Markram et al., 1997; Jia, 2022); lightweight edge networks can support millisecond-scale responses (Liu and Xu, 2025). Worst-case latency and jitter matter in addition to the mean because long-tail delays may occur precisely when movement becomes unstable or feedback is most needed.

A second challenge is learning from distributed patient populations while protecting privacy. Personalized rehabilitation models require large datasets, but institutional silos and privacy regulations limit raw data sharing. Federated learning keeps data on local devices or institutional servers and transmits encrypted model updates for aggregation. Teo et al. (2024) demonstrated that this approach can improve general models with multi-site data while allowing local fine-tuning for patient-specific variation. Federated approaches nevertheless introduce communication cost, update frequency constraints, non-independent and non-identically distributed data, privacy leakage through model updates, and model-poisoning risk. Secure aggregation, access control, version control, and auditable update policies are therefore prerequisites rather than optional add-ons.

Robust decision-making also requires multimodal fusion. Graph neural networks and Transformer architectures can represent skeletal, muscular, physiological, and temporal relationships across sensors (Zhu et al., 2024). By modeling spatiotemporal coordination, these methods may improve movement and muscle-synergy decoding under sensor displacement or partial channel failure (Mao et al., 2024). Clinical adoption, however, requires benchmark datasets, transparent error analysis, external validation, missing-channel handling, and calibrated confidence estimates. Models should degrade gracefully when input quality falls rather than force predictions from corrupted signals. Table 3 summarizes representative digital approaches, the rehabilitation decisions they support, and their key deployment and validation considerations for clinical translation. These approaches differ substantially in interpretability, computational demands, uncertainty, validation maturity, and current clinical evidence.

**TABLE 3:** Digital approaches for biosensor-informed rehabilitation decision support.

Digital approach	Decision supported	Key deployment requirements	Validation status and gaps
Edge-cloud and IoT orchestration (Xi et al., 2025)	• Continuous monitoring • Clinician dashboards • Remote escalation	• Connectivity, interoperability, and cybersecurity • Clock and version control • Encrypted transport and local fallback	• System-level validation • End-to-end reliability and fallback testing needed
Quantized edge neural networks and lightweight CNNs (Fanariotis et al., 2023; Kasper et al., 2025)	• Gait-phase recognition • Fall detection • EMG classification • Intent decoding	• Memory and battery constraints • Sensor-position shift • Long-tail latency	• Engineering and algorithmic validation • Target-population robustness and latency validation needed
Federated learning (Hinchliffe et al., 2026)	• Multi-site model development • Local personalization	• Communication cost • Secure aggregation and audited updates • Model-dependent interpretability	• Multi-site healthcare evidence • Rehabilitation-specific prospective validation and non-IID robustness needed
Graph neural networks (Mao et al., 2024)	• Multimodal movement-state interpretation • Movement and muscle-synergy decoding	• Compute load • Graph construction and missing-channel handling • Limited interpretability	• Primarily algorithmic validation • External validation and distribution-shift robustness needed
Transformers (Shang et al., 2025)	• Temporal and multimodal representation • Movement-state interpretation	• High computational and memory demands • Missing-channel handling • Limited interpretability	• Rehabilitation-specific clinical evidence limited • Calibration and external validation needed
Digital twins (Lauer-Schmaltz et al., 2024)	• Virtual testing of assistance, loading, and stimulation • Individualized recovery scenarios	• Repeated calibration and updating • Computational burden • Longitudinal data integration	• Laboratory to early-feasibility validation • Prospective clinical validation and uncertainty assessment needed
Reinforcement-learning controllers (Li et al., 2026)	• Assist-as-needed adaptation • Task-difficulty adjustment • Stimulation-parameter tuning	• Computational and training burden • Limited policy interpretability • Hard safety and fail-safe constraints	• Clinical validation remains early • Sim-to-real, safety, and controlled clinical evaluation needed
Generative AI models (Piechowiak et al., 2025)	• Synthetic pathological examples • Pretraining and stress testing	• Computational burden • Data provenance and quality control • Limited interpretability	• Primarily supportive rather than evidentiary • Real-world validation of synthetic-data utility needed

Validation status indicates translational maturity rather than methodological quality or certainty of clinical benefit.

Abbreviations: CNNs: convolutional neural networks; EMG, electromyography; IID, independent and identically distributed; non-IID, not independent and identically distributed; IoT, internet of things.

#### 3.2.2 Personalized prediction, model uncertainty, and safe adaptation

AI is shifting rehabilitation from retrospective description toward prospective and adaptive decision support. Digital twins illustrate this transition by integrating wearable gait, EMG, and biomechanical data into individualized virtual representations that can test alternative assistance, loading, or stimulation scenarios before physical application (Harlan and Tjahyadi, 2024; Piechowiak et al., 2025). Most rehabilitation digital twins remain at laboratory or early-feasibility stages because repeated calibration, model drift, uncertainty, and clinical validation remain unresolved (Olawade et al., 2026). A useful twin must update as strength, spasticity, cardiovascular tolerance, pain, and compensatory strategies change; otherwise, a static representation becomes an increasingly unreliable basis for prescription.

The main dilemma is the balance between physiological interpretability and real-time adaptability. Physics-based models offer biomechanical plausibility but can be computationally demanding, whereas data-driven models may adapt rapidly but fail under out-of-distribution pathological movement and provide limited mechanistic interpretation (Mikołajewska et al., 2024; Diniz et al., 2025). Progress toward clinical decision support will require hybrid or otherwise interpretable models, prospective multicenter evaluation, and explicit uncertainty reporting. Outputs such as estimated joint torque, physiological load, fatigue state, or fall risk are more actionable than opaque labels when they can be linked to predefined rehabilitation decisions.

Adaptive controllers extend prediction into action. Intelligent rehabilitation robots can adjust assistance, while reinforcement learning can map kinematics, EMG, fatigue, and task success to constrained changes in assistance, task difficulty, or stimulation intensity (Gu et al., 2024; Guo et al., 2024; Lauer-Schmaltz et al., 2024; Molle et al., 2026). Because autonomy increases the consequence of error, safe exploration must be bounded by pain, joint range, fatigue, charge density, and emergency-stop rules (see Box 1).

Generative models may help address data scarcity by synthesizing rare pathological examples or recovery trajectories for pretraining and stress testing (Piechowiak et al., 2025). Their role should remain supportive rather than evidentiary: synthetic data can expose algorithms to rare conditions, but sensitivity, specificity, calibration, and clinical utility must be established on prospectively collected real-world data. Greater algorithmic complexity should not be treated as synonymous with greater clinical value; a robust and interpretable model that supports a clear decision may be preferable to a nominally more accurate but opaque model whose errors are difficult to recognize.

#### 3.2.3 Biosensor-enabled biofeedback and immersive interaction

Between passive monitoring and automatic intervention lies real-time biofeedback. Integrated wearables can translate deviations in movement, loading, muscle activation, physiological tolerance, or task completion into visual, auditory, or haptic cues for self-correction or therapist-guided adaptation. Effectiveness depends on timing, salience, cognitive burden, personalization, and transfer after cue withdrawal (see Box 1).

Immersive technologies can strengthen this layer when coupled with wearable motion capture and haptics. Extended-reality tasks can turn repetitive exercise into goal-directed practice, while haptic gloves and soft actuators add tactile and proprioceptive information (Camardella et al., 2023; Lv and Singh, 2024; Tan et al., 2024; Calabrò and Morone, 2025; Chen L. et al., 2025). Evaluation should focus on practice dose, movement quality, retention, and transfer to unassisted daily activities.

Multisensory feedback may support sensorimotor engagement (Lu et al., 2025), and low-latency communication can enable remote demonstration and correction (Chaudhari, 2025). Context-aware systems may adapt assistance to behavioral context (Malathi et al., 2025), while affective-computing approaches infer stress, frustration, or fatigue (Jiao, 2025; Li and Zhang, 2025; Zakaria et al., 2025). Potential benefits must be balanced against cybersickness, overload, feedback dependence, privacy concerns, and alert fatigue; trials should therefore include real-world function, adherence, symptom burden, and caregiver burden.

These technical components become clinically meaningful only when they are connected to explicit decisions. The next section follows biosensor-derived information across assessment, biofeedback, adaptive training, therapeutic actuation, and longitudinal management.

## 4 Biosensor-informed applications across the rehabilitation continuum

Wearable applications span a continuum from low-risk monitoring to high-consequence therapeutic actuation. Evidence maturity should therefore be described by validation context: laboratory validation asks whether a signal or device works under controlled conditions; field validation tests robustness during real tasks; pilot clinical studies assess feasibility and acceptability; controlled clinical evaluation tests outcome benefit; and real-world implementation examines scalability, workload, equity, cybersecurity, and long-term safety (Table 5). The evidence threshold should rise as a system moves from measurement toward autonomous adaptation or stimulation. Accordingly, technical feasibility, immediate device-assisted performance, and clinical rehabilitation benefit are distinguished based on the outcomes directly demonstrated by the cited studies.

### 4.1 Objective assessment and continuous recovery monitoring

Wearable biosensors and complementary movement sensors support rehabilitation assessment by extracting digital measures under real-world conditions. Motor, neural, physiological, and behavioral signals can be monitored repeatedly, allowing clinicians to compare maximum capacity in the clinic with actual performance at home and to track recovery between visits. Broad deployment nevertheless depends on validity across pathological movement, sensor-placement variation, changing environments, and diverse patient populations.

#### 4.1.1 Motor, neural, and movement-function assessment

Quantifying motor and neural function is essential for individualized therapy. Traditional scales such as the Fugl-Meyer Assessment and Barthel Index remain clinically valuable but are semi-quantitative, examiner dependent, and limited to discrete timepoints (Duong et al., 2023). Table 4 compares traditional scales and wearable assessments across measurement characteristics, decision utility, and limitations. The two approaches should be viewed as complementary: clinical scales provide interpretable functional anchors, whereas wearables provide high-frequency information on exposure, symmetry, variability, context, and change over time.

**TABLE 4:** Comparison of traditional clinical scales and wearable biosensor-informed assessments in rehabilitation (Goldsack et al., 2020; Salchow-Hömmen et al., 2022; Weizman et al., 2022; Dabnichki and Pang, 2024; Daniore et al., 2024; Peng et al., 2024; Albert et al., 2025; Lieberwirth et al., 2026).

Comparison dimension	Traditional clinical scales	Wearable biosensor-informed assessments
Typical tools	• Modified Ashworth Scale • Fugl-Meyer Assessment • Barthel Index	• IMU-based gait analysis • Pressure insoles • EMG • Activity sensors • Flexible E-skin
Measurement pattern	• Discrete timepoints • Clinic-based observation	• Repeated or continuous time-series monitoring • Clinic, field, and home settings
Ecological validity	• Strong clinical familiarity • Limited observation of daily performance	• Real-world activity, variability, context, and adherence
Responsiveness	• May miss subtle or short-term changes between visits	• Recovery trajectories and day-to-day variation • Early deviation from baseline
Decision utility	• Functional anchors • Diagnostic support and established thresholds	• Movement correction and progression review • Safety monitoring and remote follow-up
Main limitations	• Inter-rater variability • Limited temporal resolution	• Placement sensitivity • Algorithm generalization • Missing data • Need for clinical validation

Abbreviations: EMG, electromyography; E-skin, electronic skin; IMU, inertial measurement unit.

This complementarity matters because a statistically precise wearable metric is not automatically meaningful to patients or clinicians. A digital biomarker should be anchored to an outcome or action: a change in gait symmetry, arm-use ratio, or step variability is useful when it indicates clinically meaningful improvement, emerging compensation, or a need to change rehabilitation dose.

Instrumented insoles, foot-mounted IMUs, and accelerometer arrays already support mature gait and activity assessment in laboratory, field, and free-living settings (Salchow-Hömmen et al., 2022; Albert et al., 2025). They can quantify spatiotemporal gait parameters, stance duration, balance, center-of-pressure trajectories, arm-use asymmetry, and day-to-day activity. Their main advantage is ecological validity: they reveal what patients actually do outside the clinic, including fatigue-related gait decline, nocturnal mobility, learned non-use, and variability in community ambulation.

Flexible electronic skin and wearable electroencephalography (EEG) extend assessment toward joint mechanics and neural intention. Soft sensors can quantify knee range of motion and angular velocity after anterior cruciate ligament reconstruction and, when combined with EMG or loading measures, may contribute to return-to-function decisions (Peng et al., 2024). Wearable EEG can detect motor-preparation signatures that behavioral tests cannot capture. These modalities remain at earlier validation stages because durability, electrode stability, placement reproducibility, and long-term home testing are limited. Multicenter use will require standardized skin preparation, attachment, calibration, and artifact handling.

Algorithmic generalizability remains a major limitation. Models trained on healthy movement may lose accuracy in people with foot supination, compensatory trunk motion, tremor, or spastic gait (Weizman et al., 2022). Representative pathological datasets and disorder-specific adaptation are therefore needed. In Parkinson’s disease, accelerometry and machine learning can quantify tremor, medication-related movement fluctuations, and freezing-of-gait risk (Dabnichki and Pang, 2024). The broader lesson is that high technical accuracy in a healthy dataset can coexist with poor clinical validity when pathology changes the signal distribution or movement strategy.

Wearable-derived metrics intended for clinical decisions should satisfy the V3 framework: verification of sensor performance, analytical validation of algorithm reliability, and clinical validation linking the metric to outcomes or states of interest (Goldsack et al., 2020; Lieberwirth et al., 2026). Clinical validation should additionally establish responsiveness to therapy and, where feasible, minimal clinically important differences. Thresholds should be linked to explicit actions such as progressing an exercise, reducing load, modifying assistance, scheduling review, or initiating a safety assessment. Without this chain, continuous sensor outputs remain exploratory data rather than actionable digital biomarkers (Daniore et al., 2024).

#### 4.1.2 Physiological tolerance, fatigue, and psychological engagement

Rehabilitation outcomes depend on the interaction among task exposure, physiological tolerance, fatigue, and engagement (Fricchione, 2023; Li and Zhang, 2025). Integrated platforms can combine internal-load measures with movement quality and task completion, helping clinicians and patients determine whether a rehabilitation dose is tolerable, insufficient, or associated with compensatory decline. This approach is particularly relevant in older or medically complex populations, where the boundary between productive overload and adverse response may be narrow.

Cardiac and pulmonary rehabilitation illustrate the value of physiological monitoring. Photoplethysmography, miniaturized electrocardiography, and pulse oximetry can track heart-rate response, rhythm, and oxygenation during exercise (Bayoumy et al., 2021; Matthews et al., 2024). Wearable ultrasound is an emerging extension that may provide repeated information on cardiac structure and hemodynamics (Ding et al., 2025). For these systems, the main challenge is not only sensing capability but maintaining contact, acoustic or optical quality, and motion tolerance during real movement.

Heart rate variability (HRV) can contribute information about autonomic state and recovery, but interpretation is context dependent and should account for posture, respiration, medication, time of day, exercise history, and individual baseline (Liu et al., 2025). A single HRV threshold should therefore not be treated as a universal trigger for changing rehabilitation intensity. More defensible adaptive systems combine physiological trends with symptoms, task performance, movement quality, and clinician-defined safety limits. Edge algorithms may then support alerts or intensity review, but claims should focus on risk detection and exercise-safety support rather than assuming direct prevention of specific adverse events (Xi et al., 2025).

Psychological state is also a determinant of adherence. Affective-computing systems combining galvanic skin response, EEG, HRV, voice, or behavioral features may estimate stress or disengagement and adapt coaching or supportive content (Jiao, 2025; Zakaria et al., 2025). Longitudinal emotion tracking is compatible with a biopsychosocial approach (Scherer, 2020), but emotional-state inference is sensitive, context dependent, and culturally variable. Meaningful consent, explainable feedback, data minimization, conservative automation, and clinician oversight are therefore essential.

### 4.2 From biosensor monitoring to individualized feedback and adaptation

Assessment becomes most valuable when it changes what happens next. Biosensor-informed rehabilitation can close the loop at four functional autonomy levels: Level 1, monitoring that triggers clinician review; Level 2, biofeedback that supports patient self-correction or therapist-guided adaptation; Level 3, automated adaptation of task difficulty or mechanical assistance; and Level 4, therapeutic actuation through FES or neuromodulation. Validation and safety requirements should increase with autonomy and the consequence of error: Level 1 requires valid measurement and clinically interpretable outputs; Level 2 additionally requires timely and usable feedback; Level 3 requires calibrated confidence, bounded control, human override, and fail-safe degradation; and Level 4 further requires end-to-end validation, physiologically appropriate timing, stimulation-specific safety limits, emergency shutdown, and clinician oversight.

#### 4.2.1 Real-time biofeedback and biosensor-guided dose adjustment

A wearable system becomes part of an adaptive pathway when a measured variable is linked to a predefined action. IMUs, pressure sensors, and EMG can identify asymmetry, timing error, excessive loading, or compensatory patterns and translate them into visual, auditory, or haptic cues. Effective feedback should be specific enough to guide correction, timely enough to relate to the task, and sparse enough to avoid annoyance, habituation, or dependence on external cues (Muthukrishnan et al., 2019; Tsiouris et al., 2020; Zhao, 2025).

Dose adjustment requires integration of more than one signal. Heart-rate response, HRV trends, oxygenation, and fatigue indicators can be combined with movement quality, task completion, and symptom reports to judge whether intensity should be progressed, maintained, or reduced (Adão Martins et al., 2021; Bayoumy et al., 2021; Liu et al., 2025). The objective is not to automate prescription from a single biomarker, but to provide a transparent evidence chain from individual baseline to current state, recommended action, and subsequent outcome.

Feedback can be directed to clinicians as well as patients. Dashboards that summarize trajectory, signal quality, adherence, and threshold crossings can support asynchronous review, whereas rapid alerts should be reserved for predefined high-risk events. This distinction reduces alert fatigue and aligns computational latency with clinical need: posture coaching may require second-scale feedback, dose review can be asynchronous, and intention-triggered stimulation may require millisecond-scale determinism.

Table 5 summarizes representative biosensor-informed decision loops across rehabilitation contexts, highlighting their sensor inputs, supported actions, outcome targets, validation gaps, and corresponding validation stages. The same monitoring–interpretation–feedback–adaptation–outcome logic applies across populations, but the variable being monitored, the person receiving feedback, the degree of automation, and the acceptable consequence of error differ substantially.

**TABLE 5:** Biosensor-informed decision loops across rehabilitation contexts.

Rehabilitation context	Biosensor inputs and derived state	Decision, feedback, or adaptation	Outcome targets	Principal validation gaps	Validation stage
Post-stroke gait rehabilitation (Chaikho et al., 2022; Porciuncula et al., 2023; Hinton et al., 2024)	• IMU, pressure, and EMG • Gait phase, asymmetry, propulsion, and muscle activation	• Haptic or visual feedback (L2) • Assistance timing (L3) and FES triggering (L4)	• Walking efficiency • Community mobility	• Generalizability to pathological gait • Sensor synchronization	• Field validation to clinical feasibility
Musculoskeletal rehabilitation (Ananías et al., 2024; Ptaszyk et al., 2025; Jin et al., 2026)	• IMU, pressure/strain, and EMG • Range of motion, loading symmetry, and movement quality	• Exercise correction (L2) • Load progression • Return-to-function review (L1)	• Functional recovery • Movement confidence • Reduced reinjury risk	• Placement variation • Task specificity • Sensor hysteresis	• Field validation to clinical feasibility
Cardiopulmonary rehabilitation (Bayoumy et al., 2021; Carmo et al., 2024; Bakhshayesh et al., 2025)	• PPG/ECG, SpO_2_, and respiratory sensing • Heart-rate response, HRV, oxygenation, and exercise tolerance	• Exercise-intensity review (L1) • Safety alerts (L1-L2) • Clinician escalation (L1)	• Safe dose progression • Improved endurance • Adherence	• Context-dependent confounding • Motion artifacts • False alarms	• Field validation to clinical feasibility
Older adults and fall prevention (Nairn et al., 2025; Ou et al., 2025)	• IMU and smart insoles • Balance, gait variability, and instability events	• Balance feedback (L2) • Task-difficulty adjustment (L3) • Safety review (L1)	• Mobility • Participation • Reduced fall risk	• Free-living false alarms • Long-term adherence • Sensor-placement variability	• Field validation to clinical feasibility
Home-based rehabilitation (Bober et al., 2024; Bakhshayesh et al., 2025; Te Boekhorst et al., 2025)	• Activity, task-completion, and physiological sensing • Adherence, fatigue, and recovery trajectory	• Automated coaching (L2) • Scheduled review (L1) • Clinician escalation (L1)	• Continuity of care • Improved access • Self-management	• Digital literacy • Data overload • Equity	• Clinical feasibility to real-world implementation
Closed-loop assistive therapy (Topalovic et al., 2023; Jin et al., 2024; Leerskov et al., 2024)	• EEG/EMG, kinematics, and impedance • Motor intention, fatigue, and state confidence	• Assist-as-needed adaptation (L3) • FES (L4) • Neuromodulation (L4)	• Task performance • Motor recovery • Participation	• Latency and artifacts • Model-confidence calibration • Fail-safe control	• Laboratory validation to clinical feasibility

Autonomy levels refer to the function performed rather than the rehabilitation context: L1, clinician-reviewed monitoring; L2, patient-facing or therapist-guided biofeedback; L3, automated adaptation of task difficulty or mechanical assistance; and L4, therapeutic actuation through FES, or neuromodulation. Validation stage reflects translational maturity rather than methodological quality, risk of bias, or certainty of clinical benefit; validation-stage ranges indicate heterogeneity in translational maturity across the cited studies.

Abbreviations: ECG, electrocardiography; EEG, electroencephalography; EMG, electromyography; FES, functional electrical stimulation; HRV, heart rate variability; IMU, inertial measurement unit; PPG, photoplethysmography; SpO_2_, peripheral capillary oxygen saturation.

#### 4.2.2 Wearable-assisted motor training and assist-as-needed adaptation

Therapist-administered training can be limited by time, labor, and the ability to deliver high-repetition practice. Sensor-integrated soft exoskeletons use fabrics, Bowden cables, or pneumatic actuators to provide assistance while preserving more natural movement than rigid frames in selected tasks (Schwartz et al., 2021; Zhang et al., 2024; Guo et al., 2025). Evidence ranges from laboratory prototypes and pilot feasibility studies to mature commercial rigid systems. Clinically relevant performance includes not only peak torque or assistance force but also donning time, comfort, skin safety, portability, battery endurance, active participation, and adaptation to day-to-day changes in spasticity and fatigue.

In post-stroke gait rehabilitation, soft exosuits can use shoe-mounted inertial sensors to detect gait phase and synchronize cable-driven assistance. Dorsiflexor assistance can improve swing-phase foot clearance, while plantarflexor assistance can improve paretic propulsion during assisted walking (Porciuncula et al., 2023). Assist-as-needed adaptation matches support to current performance to preserve voluntary effort (Hsu et al., 2024) (see Box 1); mistimed assistance may instead destabilize gait.

The motor-learning rationale is to couple voluntary intention and residual muscle activation with movement and sensory feedback while preserving active practice (Levin and Demers, 2021). EMG-based robotic rehabilitation similarly links residual muscle activation to device assistance (Huo et al., 2023). Dose should therefore be described by active repetitions, effort, assistance level, task challenge, and feedback timing rather than device-use duration.

For hand function, soft pneumatic robots use silicone chambers to assist flexion–extension while avoiding rigid-linkage injury risks (Achilli et al., 2023; Meng et al., 2023). Mirror-therapy configurations can record the unaffected hand and drive the paretic-side glove in real time, enabling bilateral cooperative training. This configuration provides temporally coupled bilateral movement and proprioceptive input, while combined soft-robotic and FES approaches have also been explored for hand-function recovery (Cardoso et al., 2022).

Combining soft exoskeletons with immersive and haptic feedback can increase repetition and engagement. Wearable haptic and multisensory systems integrate motion capture with visual, proprioceptive, and tactile feedback (Camardella et al., 2023; Chen L. et al., 2025; Lu et al., 2025). The key question is whether gains persist and transfer to unassisted daily activities.

Soft wearable robotics has moved beyond mechanical assistance toward adaptive motor learning. Clinical translation nevertheless requires standardized outcomes, long-term comfort and interface data, safety testing during unsupervised use, transparent sensor-to-actuator rules, and evidence that improvement persists after device removal. Future trials should report conventional clinical outcomes together with wearable-derived metrics such as symmetry, variability, home activity, and persistence of change after washout.

Soft exoskeletons and wearable robots primarily adapt mechanical assistance around voluntary movement; therapeutic actuation loops attempt to couple decoded state more directly to electrical or neural stimulation.

#### 4.2.3 Biosensor-triggered therapeutic actuation and neuromodulation

Closed-loop neuromodulation integrates central intention decoding with peripheral stimulation to restore sensorimotor coupling (Jia, 2022). Brain–computer interface (BCI) decode motor intent from cortical signals, whereas FES activates peripheral nerves or muscles and generates proprioceptive feedback (Sinha et al., 2021; Chen S. et al., 2025). Figure 3 conceptually summarizes the proposed intention-driven closed-loop pathway: wearable neural signals undergo local signal-quality assessment, intention decoding, and safety evaluation; the resulting FES-triggered movement and temporally aligned visual and proprioceptive feedback may reinforce associative neuroplasticity and potentially contribute to motor recovery. Intention-triggered BCI–FES systems have reached patient-facing clinical-study stages, whereas more adaptive closed-loop BCI systems that adjust feedback in real time remain at earlier stages of clinical validation (Ma et al., 2025). The sensing challenge is substantial because weak cortical signals, muscle artifact, stimulation artifact, and changing electrode impedance must be separated in real time.

**FIGURE 3:**
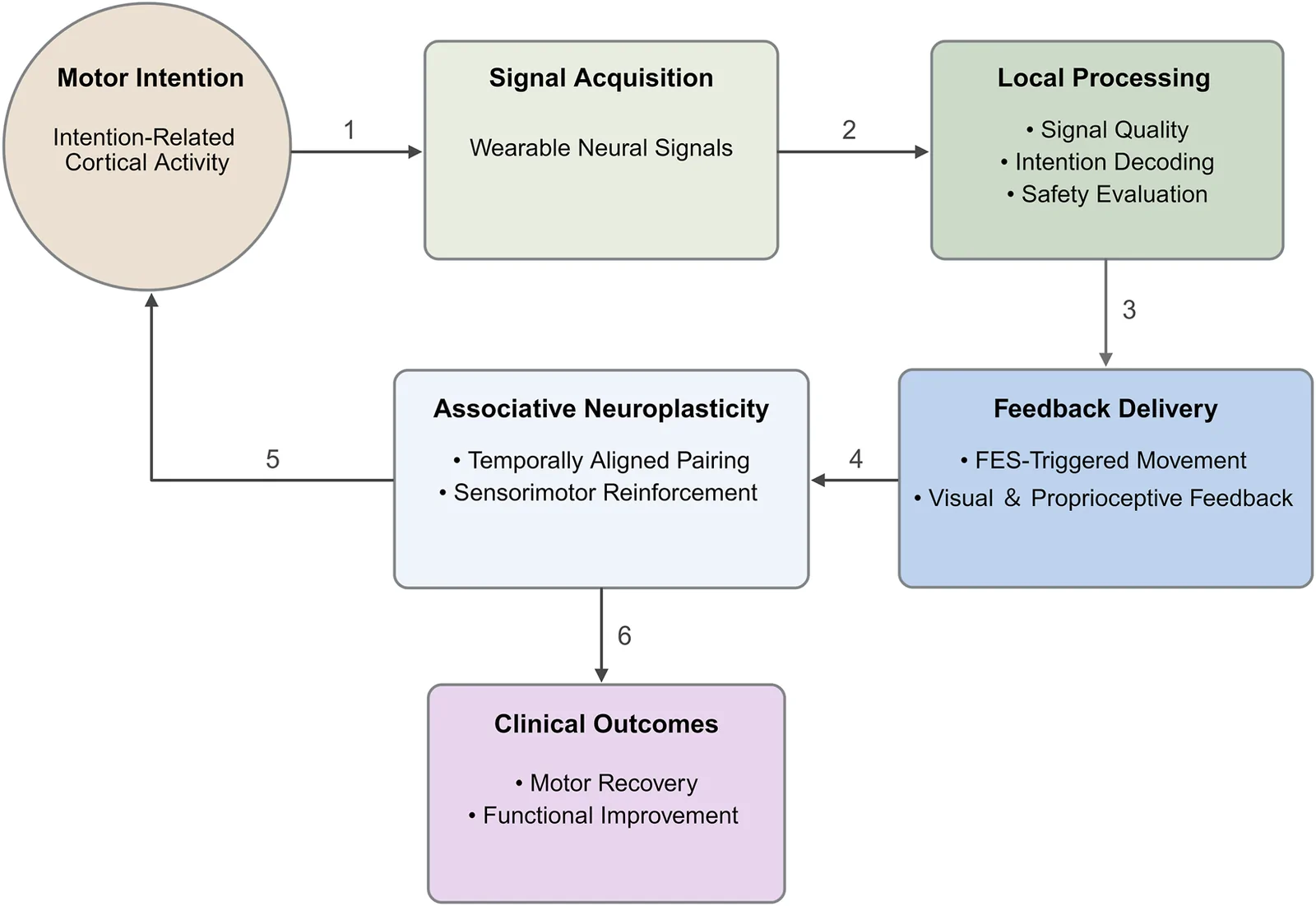
Conceptual biosensor-informed closed-loop neuromodulation pathway (motor intention → wearable neural-signal acquisition → local signal-quality assessment, intention decoding, and safety evaluation → FES-triggered movement with visual and proprioceptive feedback → temporally aligned associative neuroplasticity). The reinforced loop returns to motor intention and may support mechanisms associated with motor recovery and functional improvement. The pathway is conceptual; arrows indicate proposed or biologically plausible relationships and should not be interpreted as evidence that each transition has been established as causal in clinical rehabilitation. Created by the authors.

Artificial neural bypass architectures decode motor-intention features from EEG and trigger FES to evoke movement and sensory afference, completing the loop (Ren et al., 2024). For associative Hebbian or spike-timing-dependent plasticity–like mechanisms, intent, evoked movement, and feedback must be temporally coordinated (Markram et al., 1997; Jia, 2022) (see Box 1). Corticomuscular coupling provides a complementary measure of central–peripheral coordination (Zhang et al., 2023), although its role as a real-time control signal remains under investigation. This timing requirement favors local, deterministic triggering over cloud-dependent actuation. Because stimulation artifacts can contaminate subsequent neural recordings, blanking windows, artifact subtraction, and electrode-layout optimization are part of the core sensing design.

The frontier is shifting from unidirectional triggering to state-responsive bidirectional control. Topalovic et al. (2023) demonstrated a wearable platform that delivers stimulation contingent on decoded activity while recording single-neuron activity and local field potentials from deep structures. Such feedback allows insufficient excitability to trigger parameter augmentation and hyperactivity to trigger inhibitory modulation. These systems have high mechanistic value but remain early-stage because long-term safety, standardized control architectures, and broad clinical validation are lacking. The field should avoid conflating such mechanistic demonstrations with clinically validated rehabilitation products; their translational value depends on proving repeatability, user tolerance, and outcome benefit in target populations.

AI-driven parameter adaptation may help FES accommodate changing fatigue, pain thresholds, and excitability. Reinforcement-learning calibration can optimize frequency, pulse width, and amplitude within predefined comfort and safety constraints (Borda et al., 2023), while EEG-based algorithms may track central fatigue (Jin et al., 2024). These approaches remain at early feasibility stages; prospective trials must demonstrate comparative benefit, and controllers must enforce hard limits on charge density, duty cycle, joint range, pain, and fatigue.

Safety is decisive. Wearable closed-loop systems are vulnerable to sensor saturation, wireless dropouts, electrode detachment, and misclassification cascades (Chaikho et al., 2022); false intent detection may trigger inappropriate FES and cause fatigue, instability, or tissue injury (Borda et al., 2023; Jin et al., 2024). Fail-safe design should combine hardware current limits, impedance monitoring, signal-quality and confidence thresholds, local fallback operation, and graceful degradation to monitoring or shutdown when evidence becomes unreliable (Cousin et al., 2021). Evaluation should report intent-detection errors, fault-detection latency, shutoff time, and behavior under degraded signals, with bench tests for disconnection, electrode drying, packet loss, and movement artifacts before patient trials.

#### 4.2.4 Posture correction and daily-life assistance

Rehabilitation continues outside treatment rooms. Maladaptive posture, compensatory gait, brace non-adherence, and occupational strain may be missed during routine visits but contribute to secondary injury. Wearable biofeedback can translate biomechanical or physiological deviations into haptic, auditory, or visual prompts for self-correction during daily life (Tsiouris et al., 2020; Zhao, 2025). Although lower risk than FES or invasive neuromodulation, effectiveness still depends on timing, cue salience, habituation, and acceptance.

For Parkinsonian postural instability, IMUs embedded in belts or vests can monitor trunk tilt and trigger skin vibration when deviations exceed preset thresholds. Somatosensory prompts may be less disruptive than visual or auditory cues and can guide posture correction through conditioned responses (Muthukrishnan et al., 2019); whether these effects reduce fall risk or improve participation requires clinical validation.

For adolescent idiopathic scoliosis, smart orthotics with pressure and temperature sensors quantify brace wear time and force distribution. Connected applications feed compliance data to patients and clinicians and can use rewards to reinforce wearing behavior (Haddas et al., 2023). Similar principles apply to occupational health: smart necklaces or patches can monitor cervical flexion and neck-muscle load, warning users before chronic strain accumulates (Lingampally et al., 2022; Camboim et al., 2023). Because adolescents may remove or loosen braces for comfort, objective wear-quality measurement is more informative than self-reported compliance alone.

Wearables also support communication and social rehabilitation. Gloves combining inertial and electromyographic sensors can translate sign language into speech, functioning both as assistive communication devices and as language-rehabilitation tools for people with hearing impairment (Rodgers et al., 2019; Kudrinko et al., 2021). These applications highlight that rehabilitation outcomes include participation and quality of life, not only impairment scores. Translation will require testing under naturalistic speech, gesture speed, cultural sign-language variation, and long-term comfort constraints.

Posture and daily-assistance systems highlight an important behavioral-design principle: feedback that is too frequent can cause annoyance and abandonment, whereas feedback that is too subtle may not change behavior. Adaptive cue scheduling, individualized thresholds, and outcome measures that capture fall risk, pain recurrence, participation, and adherence are needed to move these systems beyond wellness monitoring.

#### 4.2.5 Sensor-rich smart prosthetics

For individuals with limb loss, wearable bioelectronics must go beyond assistance to reconstruct bidirectional sensorimotor function.

Next-generation prosthetics are evolving from passive mechanical replacements toward adaptive, sensory-rich, and neurally integrated systems. Commercial myoelectric and microprocessor-controlled prostheses with sensor-based gait adaptation represent the most mature end of this spectrum. Non-invasive tactile or proprioceptive feedback systems are mainly at feasibility and early clinical-evaluation stages, whereas invasive BCI prosthetic control remains constrained by safety, durability, and scalability (Jiang et al., 2023; Malešević and Antfolk, 2024). Distinguishing these maturity levels prevents overgeneralization from commercial prosthetic hardware to experimental neural interfaces.

Loss of sensory feedback contributes to prosthetic abandonment and phantom limb pain. Biomimetic electronic skin mounted on prosthetic fingertips or soles can measure pressure, temperature, texture, and contact dynamics; neuromorphic hardware can then encode these signals into bioelectrical pulses that stimulate residual peripheral nerves through transcutaneous or implantable interfaces (Wang et al., 2023; Donati and Valle, 2024). Such artificial feedback can improve grip-force adjustment during device use and support closed-loop control; longer-term effects on phantom pain and daily function require clinical validation. The analytical challenge resembles that of other biosensors: tactile sensors must preserve spatial resolution, dynamic range, and calibration stability despite repeated impacts, moisture, and mechanical wear.

Context-aware sensing and feedback add another layer of prosthetic control. Sensor-rich lower-limb prosthetic systems can combine state estimation with haptic feedback to support gait adaptation and user awareness (Karimi et al., 2025). Future non-invasive brain-machine control may further narrow the gap between biological and artificial limbs. Sustaining these benefits over years will require integration with longitudinal data ecosystems. For clinical translation, smart prosthetics should be evaluated not only by laboratory task success but also by abandonment rate, cognitive load, socket comfort, battery endurance, maintenance burden, and psychosocial acceptance.

### 4.3 Connected biosensor ecosystems for longitudinal and remote rehabilitation

Wearable data streams can extend rehabilitation from isolated sessions into longitudinal recovery management. Their value depends on whether high-density data are converted into interpretable trajectories, manageable clinician workflows, and patient-facing feedback that supports adherence without transferring inappropriate responsibility to the user. This requires an integrated ecosystem of devices, communication services, analytics, clinical interfaces, and governance rather than a stand-alone wearable.

#### 4.3.1 Recovery trajectories, interoperability, and data governance

A major system-level value of wearable devices is the creation of longitudinal records of movement, physiology, and behavior (Lee et al., 2025). Time-resolved trajectories can reveal recovery plateaus, deteriorating mobility, changes in exercise tolerance, or declining adherence before the next scheduled visit. Such archives should support both escalation and de-escalation: additional review may be needed when recovery stalls or safety signals emerge, whereas stable progress may justify greater autonomy or less frequent in-person follow-up.

Longitudinal analytics can identify changes that are difficult to observe during short visits, including intermittent rhythm abnormalities or declining gait stability (Zwack et al., 2023). Smart-device ecosystems may also support recovery forecasting and personalized follow-up (Son and Kwon, 2023). Predictive models should be audited for bias because very old adults, people with severe disability, rural users, and lower-income populations may be underrepresented in development datasets.

Real-world evidence generated from connected systems must be interpreted according to validation context. Framework-based real-world evidence approaches are useful for studying scalability and longitudinal outcomes (Kim M. et al., 2024). Condition-specific examples in Parkinson’s disease, anterior cruciate ligament rehabilitation, and balance rehabilitation illustrate the need to validate devices in target populations and environments (Moreau et al., 2023; Nairn et al., 2025; Ptaszyk et al., 2025). Data silos remain a major barrier. Cross-device integration requires common data models and semantic standards such as Fast Healthcare Interoperability Resources (Rauzi et al., 2024), together with sensor identity, calibration state, sampling frequency, time synchronization, signal-quality flags, algorithm version, and firmware version. Without these metadata, apparently similar streams may not be analytically comparable.

Synthetic data can enrich rare-event archives without exposing direct patient identities. Diffusion Transformer models have been used to synthesize realistic electrocardiographic and photoplethysmographic signals across arrhythmia types (Chen Z. et al., 2025). In rehabilitation, synthetic gait, EMG, or recovery trajectories may support federated training and stress testing, but outputs must be labeled, evaluated for distributional fidelity, and validated against real-sample distributions. Final sensitivity, specificity, calibration, and clinical utility must be established using prospectively collected real-world data.

#### 4.3.2 Self-management and Internet of Things-enabled remote rehabilitation

Self-management tools can shift patients from passive recipients of instructions toward active participants in rehabilitation. Visualized metrics such as step goals, joint range-of-motion trajectories, sleep patterns, and task completion can support self-efficacy and adherence (Wen et al., 2025). Gamification can further convert repetitive exercise into goal-directed challenges (Clarkson et al., 2022). The design objective should be actionable feedback with appropriate pacing—not maximal notification frequency or competition—so that users understand what to do next without being overwhelmed.

Remote rehabilitation addresses geographic and mobility barriers by linking home training with expert review. In stable populations such as chronic stroke survivors, randomized pilot evidence suggests that wearable-supported home telerehabilitation can achieve outcomes comparable to outpatient rehabilitation for selected motor and quality-of-life measures while reducing travel burden (Guo et al., 2023). Broader systematic-review evidence also supports telerehabilitation in selected post-stroke care contexts (Pitliya et al., 2025). These findings should not be generalized automatically to acute, severe, or cognitively impaired patients, for whom supervision and escalation requirements may differ.

AI-enabled platforms can triage monitoring streams by highlighting delayed recovery, abnormal physiological trends, poor signal quality, or falling adherence for clinician review, while stable progress receives lower-intensity automated guidance. Transparent triage rules, confidence thresholds, and escalation pathways are essential so that patients and clinicians understand when feedback is advisory, when review is required, and who is accountable for action.

Scalable deployment also requires infrastructure that connects heterogeneous devices with clinical workflows. The Basel Wearable Clinic model illustrates device-agnostic data intake, secure portals, standardized formats, and specialist interpretation (Badertscher and Brasier, 2025). Consumer devices and algorithms can change over time, potentially altering data continuity and biomarker validity. Version tracking, change control, recalibration policy, and post-deployment performance monitoring are therefore clinical implementation requirements, not merely information-technology tasks.

Regulatory and governance requirements should be proportionate to the consequence of error. Systems that summarize activity create different risks from systems that automatically change exercise dose or trigger stimulation, but in both cases the sensor, feature-extraction algorithm, model, communication layer, and user interface form a single clinical function. Updates to firmware, algorithms, or decision thresholds can change the meaning of a digital biomarker and should therefore be auditable. Permissioned distributed ledgers may offer tamper-evident records in multi-institutional networks, but they should be adopted only when they provide clear value over conventional secure audit trails, given latency, governance, energy, and privacy trade-offs.

Remote systems can also support continuity when in-person services are disrupted (Prvu Bettger et al., 2020; Brigo et al., 2022). Their longer-term value lies in reducing geographic inequity, enabling tiered care, and generating data for continuous improvement (Koscielniak et al., 2022; Nicolas et al., 2024). To avoid widening the digital divide, implementation must address device affordability, connectivity, digital literacy, caregiver support, and accessibility for sensory or cognitive impairment (Al-Dhahir et al., 2022; Bober et al., 2024; Falvey et al., 2024).

Remote rehabilitation should be evaluated as a service model rather than a device alone. Relevant outcomes include clinical effectiveness, safety events, adherence, clinician workload, escalation frequency, patient-reported experience, cost, privacy incidents, and equity impact (Alwadai et al., 2024). A sustainable model is likely to combine automated monitoring, proportionate patient feedback, scheduled clinician review, rapid escalation for predefined high-risk signals, and reimbursement structures that recognize the time required for data interpretation.

## 5 Discussion

### 5.1 From analytical performance to clinical validity

The evidence synthesized in this review shows that the principal translational bottleneck is not the absence of sensitive sensors but the chain of dependencies between analytical performance and decision utility. A signal must remain valid under movement, sweat, repeated placement, and home-use conditions; the model must recognize poor-quality or out-of-distribution data; the resulting estimate must map to a predefined and interpretable action; and the outcome must be reassessed after feedback or assistance is withdrawn. Failure at any stage can invalidate the entire loop even when laboratory sensitivity or classification accuracy is high (Goldsack et al., 2020; Daniore et al., 2024; Yin et al., 2024).

Clinical validation should therefore move beyond device-level accuracy. Wearable-derived metrics require verification, analytical validation, and clinical validation in target populations, together with responsiveness to treatment and links to patient-important outcomes (Goldsack et al., 2020; Daniore et al., 2024). The smallest sufficient sensor set may be preferable to maximal multimodality when it reduces calibration burden, missing data, power consumption, and user fatigue. Conversely, multimodal redundancy is justified when it improves confidence, identifies artifacts, or enables safe degradation after channel loss. Additional modalities should be added only when they provide nonredundant information that changes a clinically relevant decision—for example, by distinguishing physiological intolerance from poor movement quality, resolving uncertainty in a primary channel, or preserving safe operation after signal loss—and when this incremental decision value outweighs added calibration and synchronization, power demand, missing-data risk, and user burden (Daniore et al., 2024; Yin et al., 2024). These trade-offs should be specified against the exact rehabilitation decision rather than reported as generic technical advantages.

### 5.2 Privacy, interoperability, ethics, and regulatory readiness

Continuous biosensing also changes the ethical and regulatory character of rehabilitation. Longitudinal physiological, behavioral, and location-linked data can reveal sensitive information beyond the immediate rehabilitation purpose. Data minimization, meaningful consent, role-based access, encrypted transmission, secure aggregation, audit logs, and retention policies should be built into the architecture. Interoperability requires not only common clinical data standards but also sensor metadata, calibration history, time synchronization, and software-version traceability (Daniore et al., 2024; Teo et al., 2024). Algorithmic bias and the digital divide may otherwise shift benefit toward populations already well represented in datasets and well served by digital infrastructure. Higher-autonomy systems should be evaluated as integrated monitoring–feedback–adaptation loop with explicit human oversight, change control, post-deployment surveillance, and clearly assigned accountability (Falvey et al., 2024; Gu et al., 2024).

### 5.3 Evidence gaps and future research priorities

Future progress should prioritize four linked goals: robust measurement, transparent actionability, sustained engagement, and clinically meaningful outcomes. Hardware studies should report comfort, biocompatibility, reproducible placement, drift, recalibration burden, power availability, and failure behavior during prolonged use. Algorithm studies should report signal-quality awareness, external validation, subgroup performance, uncertainty calibration, missing-channel handling, worst-case latency, and behavior under distribution shift (Daniore et al., 2024; Gu et al., 2024; Yin et al., 2024). Clinical trials should distinguish immediate device-assisted performance from retention and transfer to unassisted activities and should measure function, participation, quality of life, safety, adherence, and clinician workload. Implementation research should evaluate cybersecurity, interoperability, cost, accessibility, and equity under real service conditions (Falvey et al., 2024; Teo et al., 2024).

### 5.4 Strengths and limitations of this narrative review

A strength of this review is its end-to-end, decision-centered synthesis across materials, biosensors, complementary biomechanical sensing, AI, feedback, and therapeutic actuation. This breadth also creates limitations. The targeted narrative search was not designed to identify every eligible study and did not include formal risk-of-bias assessment because the objective was to synthesize technological capabilities, decision pathways, and translational requirements across heterogeneous systems rather than estimate comparative effectiveness. Evidence maturity varies substantially across sensing modalities and rehabilitation populations, and several emerging technologies are represented mainly by engineering demonstrations or small feasibility studies. The framework should therefore be used to organize questions and compare translational requirements, not to imply equivalent clinical readiness across technologies.

## 6 Conclusion

Integrated wearable biosensors can support personalized rehabilitation only when they close a complete and accountable decision loop. Valid monitoring must be translated into interpretable state estimates; feedback must reach the right person at the right time; adaptation must remain proportionate to evidence, confidence, and risk; and subsequent outcomes must be reassessed. This logic applies across continuous monitoring, movement biofeedback, adaptive assistance, FES, neuromodulation, sensor-rich prosthetics, and home rehabilitation (Goldsack et al., 2020; Daniore et al., 2024).

The goal is not to measure more variables, deploy more complex models, or automate every decision. It is to integrate biosensing, AI, Internet of Things infrastructure, and clinical oversight into trustworthy systems that improve choices for patients and clinicians. Platforms that demonstrate the full chain—from robust sensing and secure data integration to proportionate action and patient-important outcome improvement—are the most likely to progress from promising prototypes toward personalized, continuous, and clinically accountable rehabilitation.

These conclusions should nevertheless be interpreted in light of limitations in the current evidence base. Evidence maturity varies considerably across sensing modalities, rehabilitation populations, study designs, and levels of system autonomy. Many emerging technologies, particularly digital twins, adaptive assistance, intention-triggered stimulation, and bidirectional neuromodulation, remain supported primarily by engineering demonstrations, short-duration evaluations, or small feasibility studies. Long-term multicenter evidence on clinical effectiveness, safety, usability, transfer to unassisted activities, and patient-important outcomes remains limited. The proposed framework should therefore be used to compare translational requirements and guide future validation, rather than to imply equivalent clinical readiness across the included technologies.
